# Effects of Noninvasive Brain Stimulation Combined With Antidepressants in Patients With Poststroke Depression: A Systematic Review and Meta-Analysis

**DOI:** 10.3389/fphar.2022.887115

**Published:** 2022-05-19

**Authors:** Jiabin Liang, Jie Feng, Jinhua He, Yong Jiang, Haoyu Zhang, Hanwei Chen

**Affiliations:** ^1^ Central Laboratory, Guangzhou Panyu Central Hospital, Guangzhou, China; ^2^ Graduate School, Guangzhou University of Chinese Medicine, Guangzhou, China; ^3^ Radiology Department, Sun Yat-sen Memorial Hospital, Sun Yat-sen University, Guangzhou, China

**Keywords:** noninvasive brain stimulation, poststroke depression, repeated transcranial magnetic stimulation, antidepressant, depression

## Abstract

**Objective:** To evaluated the efficacy and safety of noninvasive brain stimulation (NIBS) combined with antidepressants in patients with poststroke depression (PSD).

**Methods:** Seven databases were searched to identify randomized controlled trials of NIBS combined with antidepressants in the treatment of PSD based on the international classification of diseases (ICD-10) criteria and exclusion criteria. The retrieval time was from the database establishment to 31 October 2021. Two researchers independently screened the identified studies through the search strategy, extracted their characteristics, and evaluated the quality of the included literature. Cochrane Collaboration’s tool was used to assess risk of bias. RevMan 5.3 software was applied for meta-analysis.

**Results:** A total of 34 randomized controlled trials were included, involving 2,711 patients with PSD. Meta-analysis showed that the total effective rate was higher in the combined therapy than the antidepressant alone [odds ratio (OR): 4.33; 95% confidence interval (CI): 3.07 to 6.11; *p* < 0.00001]. The Hamilton depressive scale (HAMD) score was significantly lower in repeated transcranial magnetic stimulation (rTMS) (≤10 Hz) combined with antidepressant than in antidepressant alone [standard mean difference (SMD): −1.44; 95% CI: −1.86 to −1.03; *p* < 0.00001]. No significant difference was seen in rTMS (>10 Hz) combined with antidepressant versus antidepressant alone (SMD: −4.02; 95% CI: −10.43 to 2.39; *p* = 0.22). In addition, combination therapy more strongly improved the modified Barthel index (MBI) scale than antidepressants [mean difference (MD): 8.29; 95% CI: 5.23–11.35; *p* < 0.00001]. Adverse effects were not significantly different between two therapies (OR: 1.33; 95% CI: 0.87 to 2.04; *p* = 0.18).

**Conclusion:** Low-frequency rTMS (≤10 Hz) combined with antidepressants tends to be more effective than antidepressants alone in patients with PSD, and there are no significant adverse effects. In addition, combined therapy may enhance quality of life after stroke. Combination therapy with high-frequency rTMS (>10 Hz) showed no advantage in treating PSD. The transcranial electrical stimulation (TES) combined with antidepressants might be more effective than antidepressants alone, which are needed to confirm by more clinical trials since the.

## Introduction

Stroke is now the third leading cause of death worldwide (Benjamin et al., 2017). About 795,000 people in the United States experience new or recurrent stroke every year and, on average, a person has a stroke every 40 s (Benjamin et al., 2019). In addition to dyskinesia, patients with stroke often have psychological and emotional problems. One of the most common psychiatric complications of stroke is poststroke depression (PSD), which has an incidence in the first year after stroke as high as 33% (Hackett and Pickles, 2014). It severely affects the rehabilitation process after stroke and also exerts a heavy burden on patients’ family and on society. Despite their prevalence, depression and other mood-related deficits generally get the least attention. Accordingly, mood disorders need to be addressed during the rehabilitation process of stroke to improve quality of life.

Antidepressants are currently the mainstay of treatment for PSD, but certain adverse reactions are inevitable (Hackett et al., 2008; Coupland et al., 2011). For example, tricyclic antidepressants (TCAs) and selective serotonin reuptake inhibitors (SSRIs) increase the risk of cardiovascular and anticholinergic adverse effects. Fluoxetine has also been reported to be unable to improve PSD symptoms (Robinson et al., 2000; Rice et al., 2021), and some patients with stroke do not respond to antidepressants (Anderson et al., 2004; Hackett et al., 2008). Thus, an effective combination therapy for PSD is urgently required.

Repeated transcranial magnetic stimulation (rTMS) and transcranial electrical stimulation (TES) have been proven to be effective in boosting upper limb rehabilitation and improving aphasia after stroke (Vines et al., 2008; Szaflarski et al., 2011; Hu et al., 2018; Lefaucheur et al., 2020; Kuzu et al., 2021). More and more clinical trials have recently focused on noninvasive brain stimulation (NIBS) treatment of PSD (George et al., 1995; Jorge et al., 2008; Tang et al., 2018), and most have identified positive effects. We have found that the clinical effect of NIBS combined with antidepressants may be better than that of antidepressants alone, and many studies have also mentioned this possibility (Slotema et al., 2010; Brunoni et al., 2013). The current meta-analysis evaluated the efficacy and safety of NIBS combined with antidepressants in the treatment of PSD to provide evidence-based information for clinical decision-making and guideline recommendations.

## Materials and Methods

### Search Strategy

Relevant randomized controlled trials (RCTs) of NIBS combined with antidepressants in the treatment of PSD were retrieved from the following databases: PubMed, EMBASE, Web of Science, CNKI, Cochrane Library, Biology Medicine Disc (CBM), and the Wanfang database. The retrieval time was from database establishment to October 2021. Search criteria were formulated according to different databases. The keywords included “noninvasive brain stimulation,” “repeated transcranial magnetic stimulation,” “transcranial direct current stimulation,” “transcranial magnetic stimulation,” “antidepressant,” “antidepressant drugs,” “western medicine,” “after stroke,” “poststroke,” and “depression”. Only English and Chinese articles were considered.

### Inclusion Criteria

The literature included conformed to the following inclusion criteria (I) participants: patients were diagnosed with PSD and included those with ischemic stroke and hemorrhagic stroke, with no limit on the degree of depression. The diagnosis of PSD met the international classification of diseases (ICD-10) criteria for organic mental disorder (Brämer, 1988), and the score of Hamilton rating scale for depression (HAMD) exceeds 7 (Hamilton, 1960), the first onset, and the diagnosis of stroke was confirmed by magnetic resonance imaging (MRI) or computed tomography (CT), along with being down in spirits, fatigue and lack of interest. (II) study type: RCT; (III) interventions and comparisons: studies comparing the combination of noninvasive brain stimulation and antidepressants with antidepressants alone, such as fluoxetine, paroxetine, sertraline, fluvoxamine, citalopram, maprotiline, imipramine, amitriptyline, doxepin, and chlorimipramine, with only rTMS and TES chosen as noninvasive brain stimulation in this analysis and no frequency limit in the rTMS; (IV) primary outcomes: total effective rate and Hamilton depressive scale (HAMD) score; and (V) secondary outcomes: adverse effect rate and modified Barthel index (MBI) scale score.

### Exclusion Criteria

Exclusion criteria of this study were as follows (I) language: non-English or non-Chinese studies; (II) study type: not RCTs, such as animal experiments, reviews, retrospective studies, case reports, conference, and comments; (III) duplicate records, those with incomplete, unclear or inconsistent outcomes, or those with missing information that could not be obtained from the authors; and (IV) studies without a control group or with placebo stimulation or NIBS at a different frequency to the control group.

### Data Extraction and Management

Two researchers independently searched and browsed the databases according to the retrieval strategy and then carefully read the full article and extracted the characteristics of the included literature. The following information on the included literature was recorded: authors’ names, publication year, sample size, participant age, intervention, control, outcome indicators, and stimulation frequency, intensity, orientation, control, and duration. Any disagreements were negotiated and discussed with a third researcher.

### Quality Assessment

Cochrane Collaboration’s risk of bias tool was used to assess the quality of the included studies. The tool considers six items: selection bias, performance bias, detection bias, attrition bias, reporting bias, and other biases. Each item was judged as one of three levels: low risk, unclear risk, or high risk.

### Statistical Analysis

We used RevMan 5.3 software to perform this meta-analysis. The weighted mean difference was used for continuous variables, whereas the odds ratio (OR) was used for dichotomous variables. All data were calculated with 95% confidence intervals (95% CIs). Heterogeneity analysis and sensitivity analysis were also performed using RevMan 5.3. The random-effects model was selected if significant heterogeneity was identified (*p* < 0.05 or I^2^>50%). Subgroup analysis and investigation of heterogeneity in subgroups were conducted when necessary. The fixed-effects model was selected if the heterogeneity was low (*p* ≥ 0.05 or I^2^ ≤ 50%). Reporting bias was assessed by funnel plot, with dissymmetry indicating significant reporting bias in the analysis.

## Results

### Selection of Results

**FIGURE 1:**
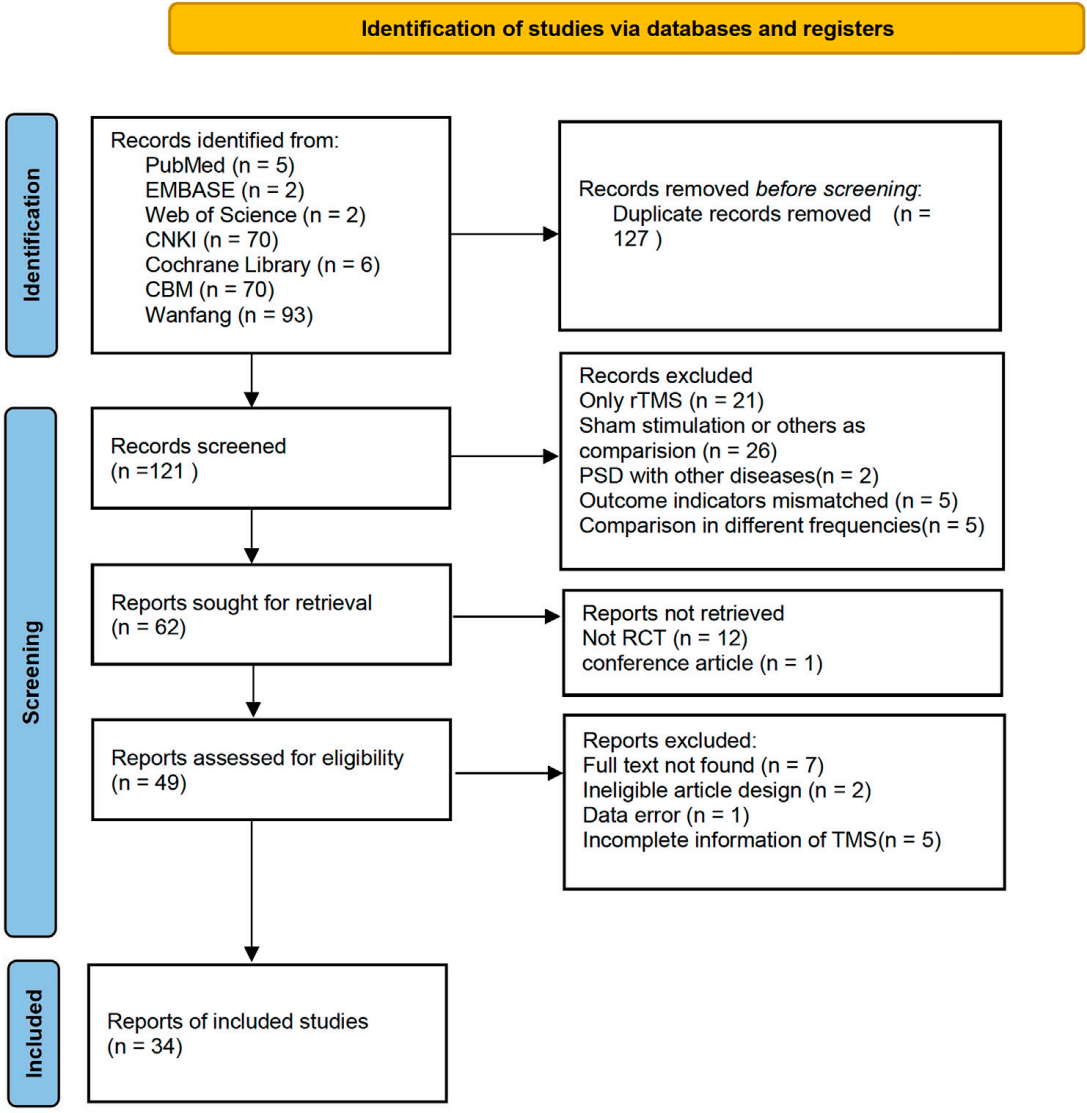
Flow diagram of article selection.

A total of 555 records were identified in the electronic databases. Of these, 248 records remained after the two researchers read the titles. After the deletion of duplicates and exclusion of 71 studies due to inconsistent primary standards after abstract screening, the full text of 50 articles were read for further assessment. Finally, 34 studies were selected for analysis. Figure 1 shows the flow diagram of the article selection.

**FIGURE 2:**
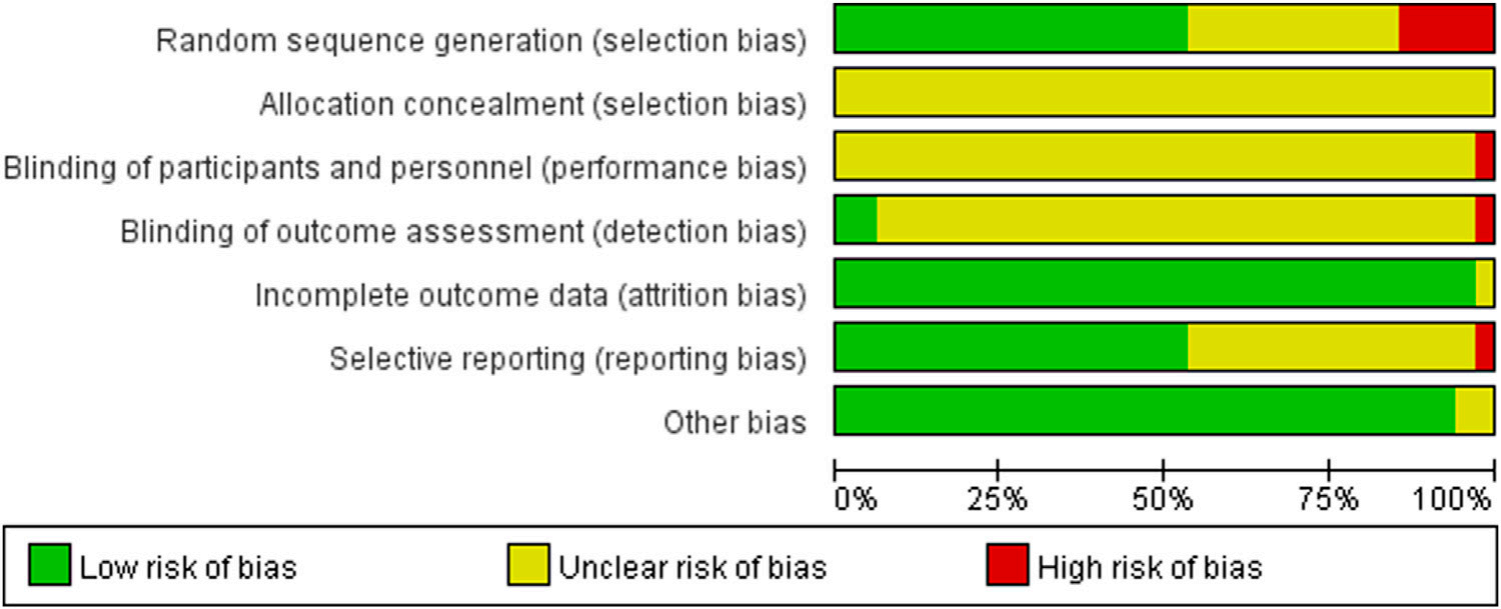
Risk of bias.

**FIGURE 3:**
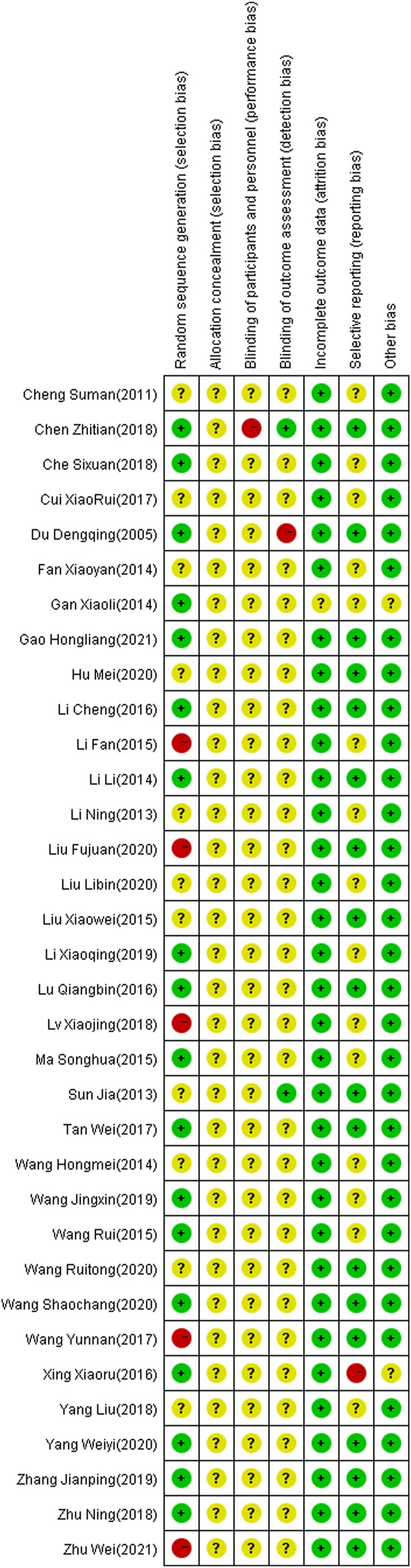
Summary of risk of bias of included studies. Red, high risk; green, low risk; yellow, unclear risk.

The 34 selected studies (Sun and Song, 2013; Hm, 2014; Ma and Ma, 2015; Wang and Ding, 2015; Xing and Wang, 2016; Tan and Zhou, 2017; Wang and Wen, 2017; Yang and Shi, 2018; Zhu, 2018; Wang and Li, 2019; Zhang, 2019; Wang and Qin, 2020; Wang and Wu, 2020; Yang and Hu, 2020; Wei, 2021) included a total of 2,784 patients, with 1,391 patients in the NIBS combined with antidepressant group and 1,393 patients in the antidepressant alone group. The basic characteristics of the included studies are summarized in Supplementary Table S1, including the authors’ names, publication year, sample size, participant age, type of stroke, intervention, control, outcome indicators, and stimulation frequency, intensity, orientation, control, and duration. There was no significant difference in the baseline data between the two groups. The quality assessment of the included studies is shown in Figures 2, 3.

### Meta-Analysis Results

The main indicators were the HAMD score and the total effective rate after treatment. The secondary outcome indicators were the MBI score and adverse effects after treatment.

#### HAMD Score

**FIGURE 4:**
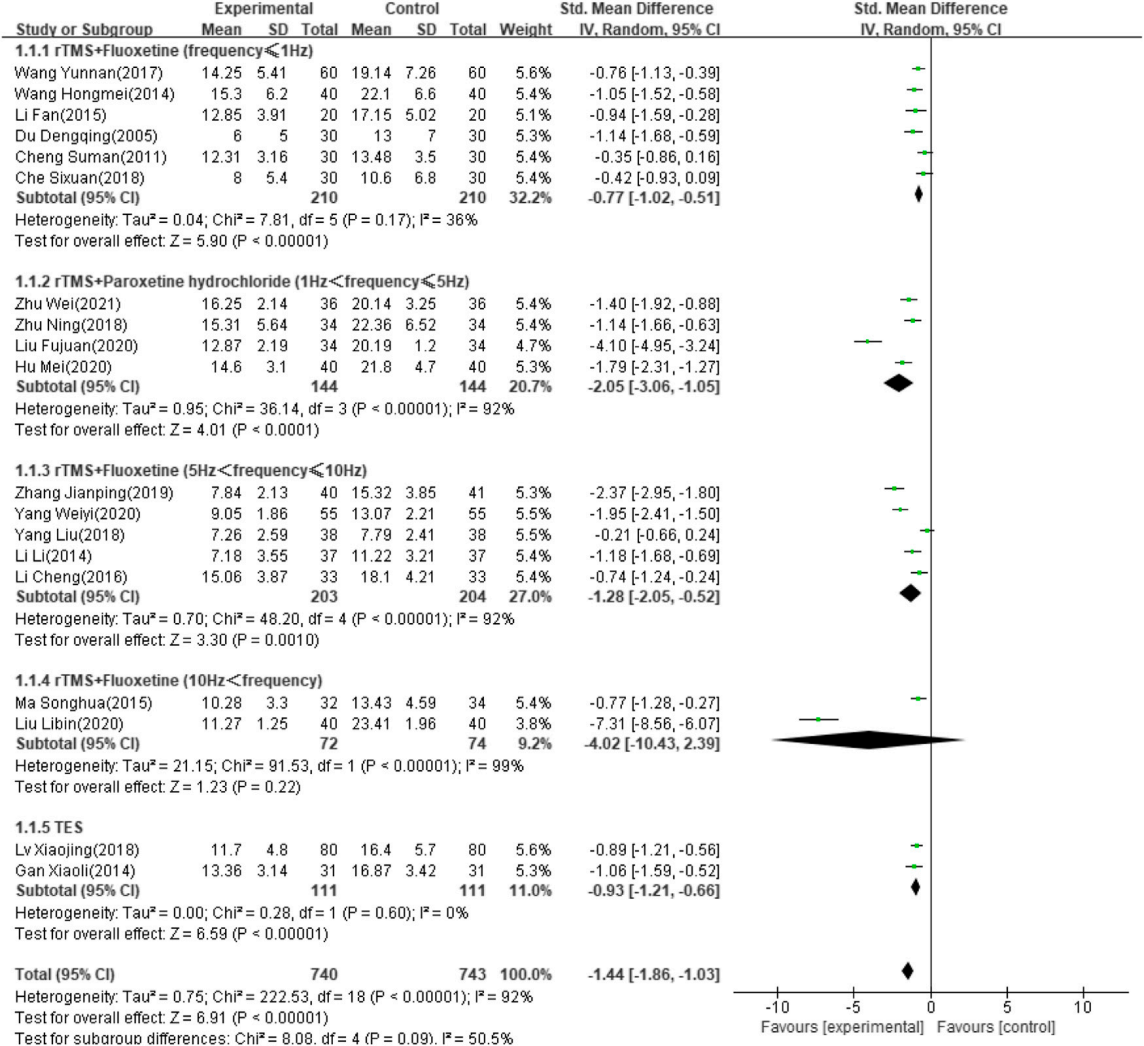
Forest plot of HAMD score.

Thirty-four of the selected studies (Wei, 2021; Zhu, 2018; Zhang, 2019; Yang and Hu, 2020; Yang and Shi, 2018; Xing and Wang, 2016; Wang and Wen, 2017; Wang and Qin, 2020; Wang and Wu, 2020; Wang and Ding, 2015; Wang and Li, 2019; Hm, 2014; Tan and Zhou, 2017; Sun and Song, 2013; Ma and Ma, 2015; Xj, 2018; Lu and Yang, 2016; Liu, 2015; Liu and Wang, 2020; Li and Chen, 2019; Li and Pan, 2013; L, 2014; Li, 2017; Li and Liang, 2016; Hu and Chen, 2020; Hl, 2021; Gan and Wang, 2015; Xy, 2014; Du, 2005; Xr, 2017; Cheng, 2011; Tian, 2018; Che and Chang, 2018), which involved 2,711 patients, reported the HAMD score as an outcome indicator. Because heterogeneity test analysis showed that there was significant heterogeneity among the included articles (I2 = 96%, *p* < 0.00001), a random-effects model was used to combine results. Subgroup analysis was performed according to intervention frequency and antidepressant category (Zhu, 2018; Zhang, 2019; Yang and Hu, 2020; Yang and Shi, 2018; Xj, 2018; Wei, 2021; Wang and Wen, 2017; Ma and Ma, 2015; Liu and Wang, 2020; Li, 2017; Li and Liang, 2016; L, 2014; Hu and Chen, 2020; Hm, 2014; Gan and Wang, 2015; Du, 2005; Cheng, 2011; Che and Chang, 2018; Fj, 2020). The meta-analysis showed that the difference was significant (SMD: −1.44; 95% CI: −1.86 to −1.03; *p* < 0.00001) (Figure 4). However, studies using rTMS combined with fluoxetine with a frequency exceeding 10 Hz showed no significant effect after treatment (SMD: −4.02; 95% CI: −10.43 to –2.39; *p* = 0.22).

#### Total Effect Rate

**FIGURE 5:**
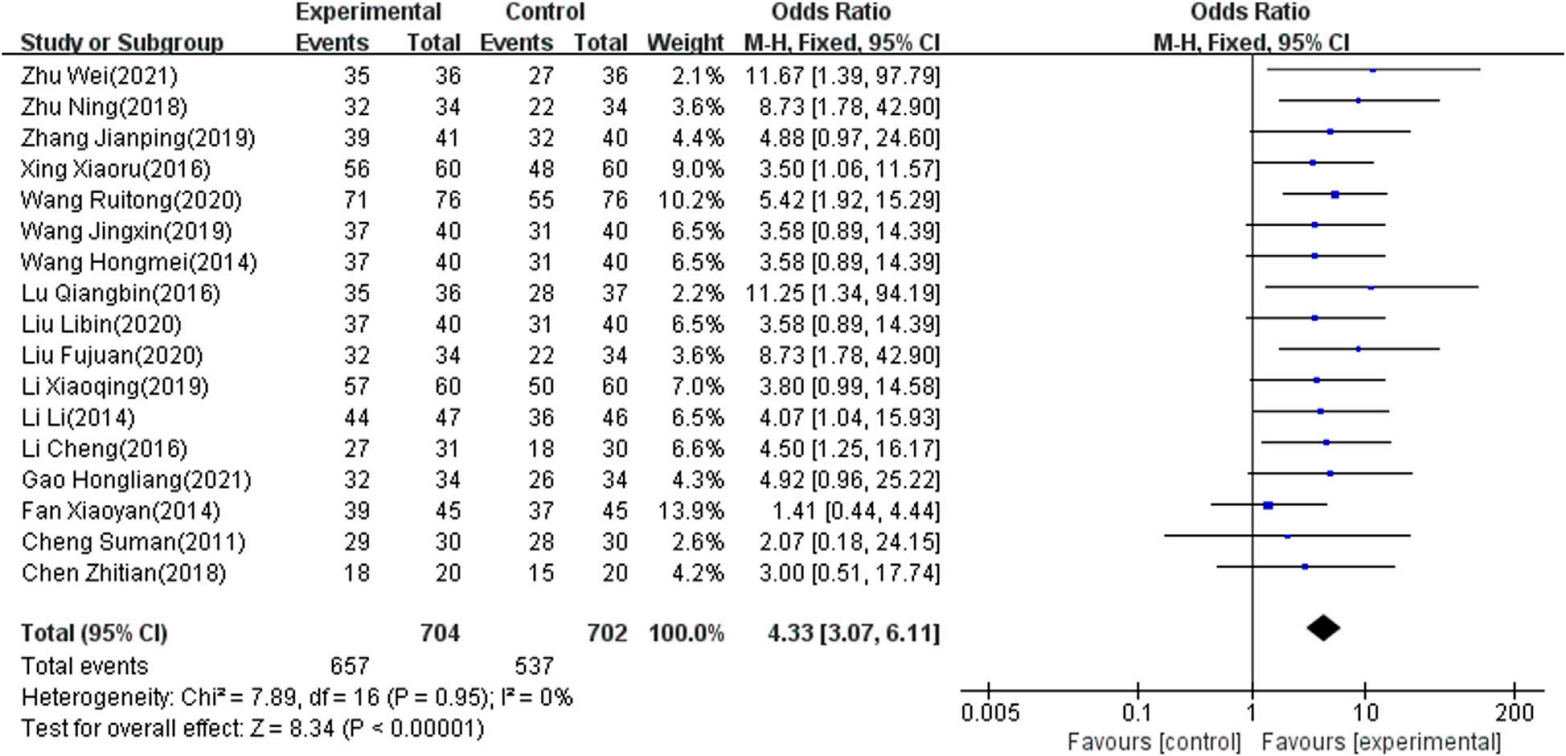
Forest plot of total effect rate.

Seventeen of the included studies (Zhu, 2018; Zhang, 2019; Xy, 2014; Xing and Wang, 2016; Wei, 2021; Hm, 2014; Wang and Qin, 2020; Wang and Li, 2019; Tian, 2018; Lu and Yang, 2016; Liu and Wang, 2020; Li and Chen, 2019; Hl, 2021; Li and Liang, 2016; Cheng, 2011; L, 2014; Fj, 2020), which involved 1,406 patients, reported the total effect rate as an outcome indicator. There was significant heterogeneity among the included articles (I2 = 0%, *p* < 0.00001). Accordingly, the fixed-effects model was used to combine results. The meta-analysis showed that the difference was significant (OR: 4.33; 95% CI: 3.07 to 6.11; *p* < 0.00001) (Figure 5).

#### MBI Score

**FIGURE 6:**
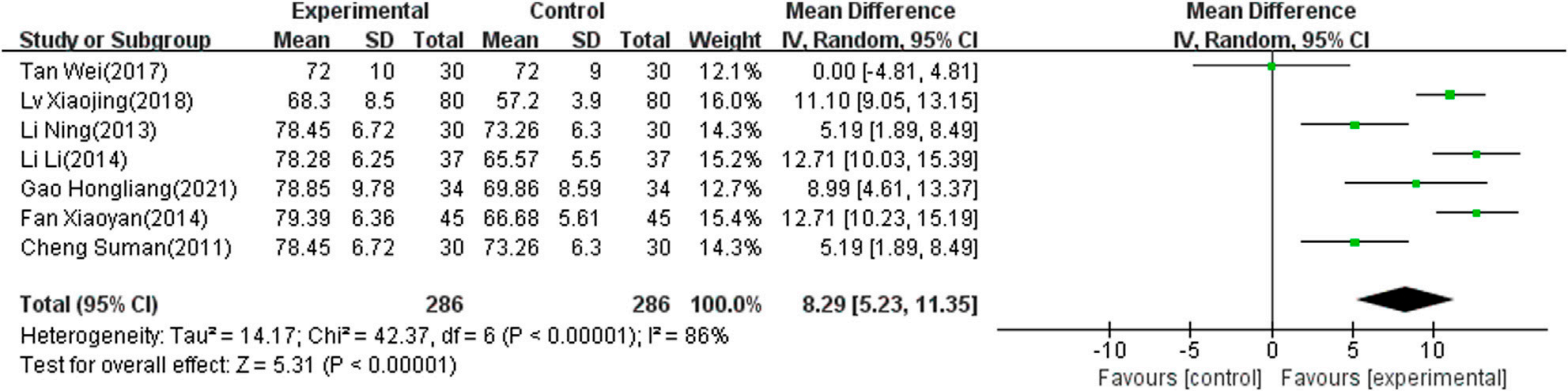
Forest plot of MBI score.

Seven of the selected studies (Xy, 2014; Xj, 2018; Tan and Zhou, 2017; Li and Pan, 2013; L, 2014; Hl, 2021; Cheng, 2011), involving 572 patients, reported the MBI score as an outcome indicator. The included articles showed significant heterogeneity (I2 = 86%, *p* < 0.00001) and the random-effects model was therefore used to combine results. The meta-analysis showed that the difference was significant (MD: 8.29; 95% CI: 5.23 to 11.35; *p* < 0.00001) (Figure 6).

#### Adverse Effect Rate

**FIGURE 7:**
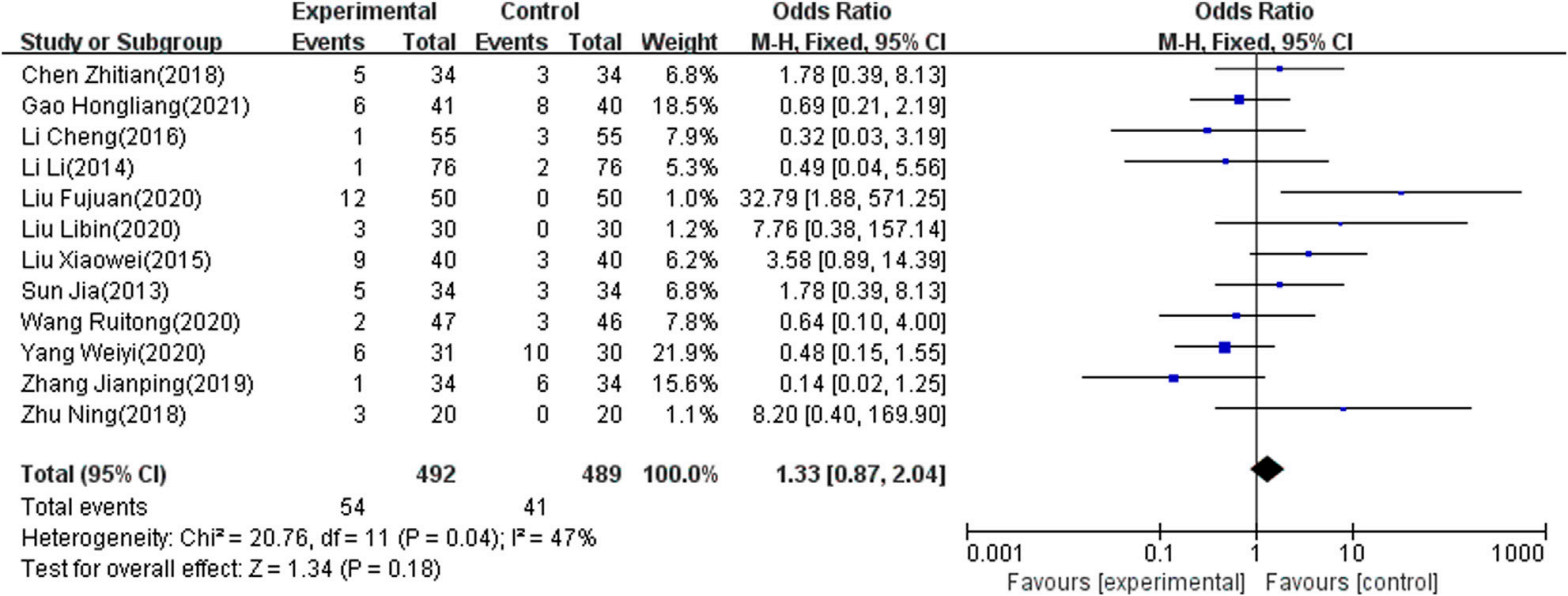
Forest plot of side effect rate.

The adverse effect rate was reported as an outcome indicator in 12 of the included studies (Zhu, 2018; Zhang, 2019; Yang and Hu, 2020; Wang and Wu, 2020; Tian, 2018; Sun and Song, 2013; Liu, 2015; Liu and Wang, 2020; Li and Liang, 2016; L, 2014; Hl, 2021; Fj, 2020), which involved 981 patients. Because heterogeneity test analysis showed that there was significant heterogeneity among the included articles (I2 = 47%, *p* = 0.04), the fixed-effects model was used to combine results. The meta-analysis showed that there was no significant difference in the adverse effect rate between the two groups (OR = 1.33; 95% CI: 0.87–2.04, *p* = 0.18) (Figure 7).

Adverse reactions mainly included behavioral toxicity, nervous system abnormalities, and cardiovascular system abnormalities. Behavioral toxicity included somnolence and epilepsy. Nervous system abnormalities commonly included headache. Digestive system abnormalities included nausea, vomiting, and indigestion. In the NIBS combined with antidepressant group, 36 patients had headache, three had insomnia, three had thirst, eight had nausea, 12 had vomiting, and two had cardiovascular system abnormalities. In the antidepressant group, four patients had headaches, three had insomnia, four had thirst, five had nausea, 13 had vomiting, one had fatigue, and one had cardiovascular system abnormalities.

#### Sensitivity Analysis

Sensitivity analyses of each outcome indicator were performed by excluding single articles one-by-one to test the effect of each study on the pooled effect size. In the meta-analysis of the HAMD score, the heterogeneity decreased from 92% to 34% after deleting the study by Liu FJ from 2020 (Fj, 2020). The results showed that this heterogeneity was mainly due to this study. There was no qualitative change in the combined effect for all outcome indicators. Thus, the pooled results of the included studies were steady.

#### Publication Bias

**FIGURE 8:**
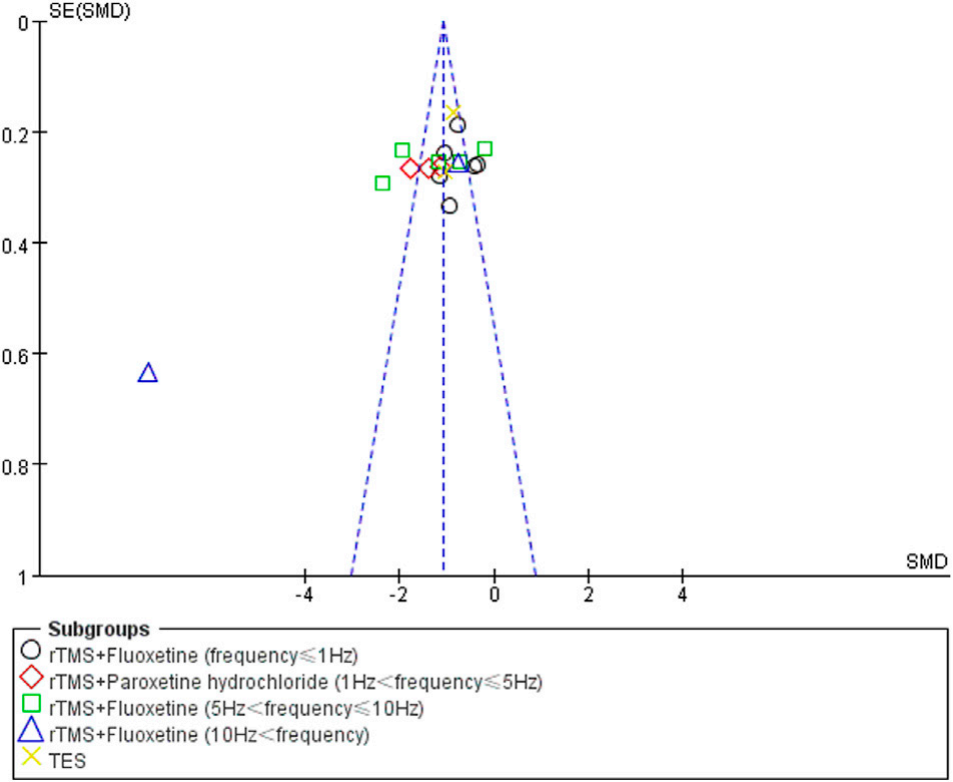
Funnel plot of publication bias. SE, standard error; SMD, standard mean difference.

Funnel plot analysis was used to analyze the publication bias of the HAMD score, total effect rate, and adverse effects. There was no obvious publication bias in the studies of the total effect rate and adverse effects. The poor symmetry of the funnel plot indicated the existence of a publication bias due to the study by Liu LB from 2020 (Liu and Wang, 2020). After deleting this study, the combined effect was not changed but the total heterogeneity decreased to 79%. The publication bias results for the HAMD score analysis are shown in Figure 8.

## Discussion

Our meta-analysis included 34 studies of the effects of NIBS combined with antidepressants for patients with PSD. The results showed that the combination of NIBS and antidepressants might have a better effect on PSD and could improve the depression scale score and quality of life compared with antidepressants alone. It is well known that guidelines recommend rTMS for the treatment of major depression, and many meta-analyses have shown that TMS intervention with PSD was positive (Shen et al., 2017; Liu et al., 2019; Shao et al., 2021), but a growing number of studies have recommended multi-module combination therapy and population-specific personalized treatment (Wang et al., 2019; Nestor and Blumberger, 2020), which warrants further research on the frequency and site of TMS intervention. The use of low-frequency TMS by Daniel R Schaffer significantly improved depression with cognitive impairment, suggesting that low-frequency TMS is more effective in specific populations (Schaffer et al., 2021). Compared with previous reviews (Bucur and Papagno, 2018; Liu et al., 2019), our analysis had the following advantages (I) combination NIBS and antidepressant therapy; (II) internationally recognized depression assessment scales; (III) inconsistent results with previous studies due to negative outcomes of high-frequency TMS combined with antidepressants; and (IV) the inclusion of more than 30 studies. There have been no studies evaluating NIBS in combination with antidepressants for PSD, although combination therapy is more clinically appropriate. Therefore, this meta-analysis may have a greater reference value than previous reviews.

According to the results of this analysis, combined NIBS and antidepressant therapy reduced the HAMD score of PSD more than antidepressants alone. However, this result was highly heterogeneous. We grouped the studies by a variety of clinically relevant factors, including age, intervention frequency and intensity, drug type, type of stroke, and stimulation orientation and duration. In the final analysis, rTMS combined with fluoxetine (less than 1 Hz and between 5 and 10 Hz) was more effective than fluoxetine alone, but the effect was not better with a frequency exceeding 10 Hz. TES combined with antidepressants improved the HAMD score more than antidepressants alone, although only two included articles examined this combination. After deleting the study by Liu FJ from 2020 (Fj, 2020) due to its high heterogeneity, rTMS combined with paroxetine was also more effective in reducing the HAMD score. This previous study by Liu FJ (Fj, 2020) was probably a retrospective study due to its vague description and was excluded from the pooled effect. Martijn (Arns et al., 2010) also commented that there were possibly differential effects of different rTMS stimulation frequencies, although many searches concluded that high-frequency rTMS has the same effect as low-frequency rTMS or antidepressants (Berlim et al., 2013).

The results of 17 studies (Zhu, 2018; Zhang, 2019; Xy, 2014; Xing and Wang, 2016; Wei, 2021; Wang and Qin, 2020; Hm, 2014; Hl, 2021; Wang and Li, 2019; Tian, 2018; Lu and Yang, 2016; Liu and Wang, 2020; Li and Chen, 2019; Li and Liang, 2016; Cheng, 2011; L, 2014; Fj, 2020) also indicated that NIBS combined with antidepressants was better than antidepressants alone regarding the total effect rate. Moreover, for the MBI score, seven studies (Xy, 2014; Xj, 2018; Tan and Zhou, 2017; Li and Pan, 2013; L, 2014; Hl, 2021; Cheng, 2011) showed that the combination therapy has potential benefits in patients with PSD. Combination therapy may be able to improve quality of life after stroke. Since a few included articles reported MBI scores, the meta-regression did not be conducted. Subgroup analyses were added based on clinical characteristics, including frequency, intensity and location of intervention, degree of depression, and course of disease. Heterogeneity still could not decrease to a reasonable range. We used sensitivity analysis to find no articles causing high heterogeneity, and adopted a random effect model. This result is stable and conservative. Some studies (Zhu, 2018; Zhang, 2019; Yang and Hu, 2020; Ma and Ma, 2015; L, 2014; Fj, 2020) have reported headache, nausea, vomiting, insomnia, thirst, and fatigue in both control and experimental groups. The adverse reactions may be caused by antidepressants. Twelve studies (Zhu, 2018; Zhang, 2019; Yang and Hu, 2020; Wang and Wu, 2020; Tian, 2018; Sun and Song, 2013; Liu, 2015; Liu and Wang, 2020; Li and Liang, 2016; L, 2014; Hl, 2021; Fj, 2020) demonstrated consistent and stable results in adverse reaction rates. This suggests that combined NIBS and antidepressant therapy is safe.

There is still a contradiction between the advantages and disadvantages of the different frequencies of NIBS, and the effects of different frequencies of NIBS are still disputed. Different frequencies of rTMS have been shown to reduce fluorodeoxy glucose F18 (^18^F-FDG) uptake in the dorsal cortical region while simultaneously increasing ^18^F-FDG uptake in the ventral region (Parthoens et al., 2016). The rTMS decreased glucose metabolism in the stimulated temporal region, with increases in the bilateral precentral, ipsilateral superior and midfrontal, prefrontal, and cingulate gyri. This suggests that 1 Hz rTMS could induce cortical regulation and extensive changes in the neural network through long-range neuronal connectivity (Lee et al., 2013). Studies have also shown that low-intensity TMS mainly stimulates low-threshold inhibitory neurons (Duan et al., 2018). High-frequency TMS caused greater activation than low-frequency TMS in normal humans. However, oxidative stress, lipid peroxidation, and protein oxidation were found in the neural tissue of stroke patients. Any of these pathophysiological processes may be related to PSD (Nabavi et al., 2015). Kimbrell et al. (1999) also reported that the antidepressant response to rTMS might depend on the pretreatment cerebral metabolism and the stimulation frequency. Thus, it is possible that patients with PSD are more sensitive to low-frequency TMS. Due to abnormal expression of amine neurotransmitters and cytokine expression after stroke, the combination of antidepressants with rTMS may be more effective with the mild stimulation of low-frequency TMS.

The mechanism of PSD is still unclear, which may involve neurobiological pathways, inflammation and apoptosis mechanisms (Robinson and Jorge, 2016; Medeiros et al., 2020). Robinson (Robinson et al., 1984; Narushima et al., 2003) suggested that lesions in the left frontal lobe or left basal ganglia were associated with PSD. And focal brain stimulation using rTMS was only effective when administered to the left dorsolateral prefrontal cortex in patients with vascular depression (Jorge et al., 2008). A meta-analysis (Carson et al., 2000) showed that stroke site was not associated with depression, and a study (Wei et al., 2015) suggested a significant association between stroke in the right hemisphere and the incidence of depression. Some studies have hypothesized that stroke lesion area is related to depression degree, which could be explained by some pro-inflammatory factors (Spalletta et al., 2006). For example, the increase of IFN-γleads to the cascade reaction of other pro-inflammatory cytokines IL-6, IL-1βand TNF-α, which aggravates depression. Secondly, IFN-γcan affect the HPA axis (Capuron et al., 2003), leading to increased adrenocortical hormone and cortisol levels, resulting in increased reactive oxides (Altieri et al., 2012; Ferrari and Villa, 2017), which further cause cell death and damage. Proinflammatory factors also stimulate the activity of indoleamine 2, 3-dioxygenase, which degrades tryptophan, the biological precursor of serotonin, into a toxic metabolite (Bansal and Kuhad, 2016). Compared with common depression, PSD is associated with focal ischemia, which leads to programmed cell death, cell swelling, or cell necrosis and a series of complex events related to cellular and molecular mechanisms (Brouns and De Deyn, 2009). Whether the neuroanatomical location of stroke affects depression remains controversial. It remains unknown whether the severity of stroke is positively correlated with the severity of depression, or whether there are differences in depression at different times after stroke. It is hoped that more RCTs will be designed in this direction in the future.

## Limitations and Prospects

This meta-analysis emphasized the clinical efficacy and depression improvement of combination therapy in PSD patients but also examined quality of life and safety. However, all included RCTs were from China, which may indicate publication bias. Funnel plot analysis revealed that a study by Liu LB (Liu and Wang, 2020) had significant publication bias due to selective reporting of outcomes. Accordingly, the result should be treated with caution. This meta-analysis was not registered and there may be a small deviation, but we still strictly followed the procedures of systematic evaluation. In addition, some indicators were significantly heterogeneous. Therefore, caution is required for these findings. More basic studies are needed to determine the mechanism underlying the effect of low-frequency TMS combined with antidepressants on depression after stroke. Moreover, large multicenter studies are needed to assess the best frequency and type of depression drugs to promote the final translation of combination treatment into daily clinical practice and guidelines.

## Conclusion

Our analysis demonstrate that low-frequency rTMS(10 ≤ Hz) combined with antidepressants tends to be more effective than antidepressants alone in patients with PSD and there are no significant adverse effects. In addition, combined therapy may boost quality of life after stroke. Combination therapy with high-frequency rTMS (>10 Hz) showed no advantage in treating PSD. The transcranial electrical stimulation (TES) combined with antidepressants may be more effective than antidepressants alone. More randomized controlled studies with detailed design for different stroke periods, depression levels and stroke location are needed to verify this conclusion.

## Data Availability

The original contributions presented in the study are included in the article/Supplementary Material, further inquiries can be directed to the corresponding authors.
